# Combinatorial brain decoding of people's whereabouts during visuospatial navigation

**DOI:** 10.3389/fnins.2013.00078

**Published:** 2013-05-20

**Authors:** Hans P. Op de Beeck, Ben Vermaercke, Daniel G. Woolley, Nicole Wenderoth

**Affiliations:** ^1^Laboratory of Biological Psychology, University of Leuven (KU Leuven)Leuven, Belgium; ^2^Motor Control Laboratory, University of Leuven (KU Leuven)Leuven, Belgium; ^3^Neural Control of Movement Lab, ETH ZürichZürich, Switzerland

**Keywords:** fMRI, pattern classification, visual cortex, motor cortex, objects and faces

## Abstract

Complex behavior typically relies upon many different processes which are related to activity in multiple brain regions. In contrast, neuroimaging analyses typically focus upon isolated processes. Here we present a new approach, combinatorial brain decoding, in which we decode complex behavior by combining the information which we can retrieve from the neural signals about the many different sub-processes. The case in point is visuospatial navigation. We explore the extent to which the route travelled by human subjects (*N* = 3) in a complex virtual maze can be decoded from activity patterns as measured with functional magnetic resonance imaging. Preliminary analyses suggest that it is difficult to directly decode spatial position from regions known to contain an explicit cognitive map of the environment, such as the hippocampus. Instead, we were able to indirectly derive spatial position from the pattern of activity in visual and motor cortex. The non-spatial representations in these regions reflect processes which are inherent to navigation, such as which stimuli are perceived at which point in time and which motor movement is executed when (e.g., turning left at a crossroad). Highly successful decoding of routes followed through the maze was possible by combining information about multiple aspects of navigation events across time and across multiple cortical regions. This “proof of principle” study highlights how visuospatial navigation is related to the combined activity of multiple brain regions, and establishes combinatorial brain decoding as a means to study complex mental events that involve a dynamic interplay of many cognitive processes.

## Introduction

The days in which brain imaging was mostly used for localization and neo-phrenology seem far behind us, if such days ever existed at all. In recent years many neuroimaging studies have tried to find out not only *where* processes and representations are implemented neurally, but also *how* these processes and representations come about and *what* their characteristics are. A powerful group of methods to find answers to such questions has been variously referred to as brain decoding, brain reading, pattern classification methods, and multi-voxel pattern analyses. Typically, brain decoding fMRI studies have determined how isolated aspects of perception and cognition can be decoded from patterns of neural activity in functionally specialized brain regions, in cases where the occurrence of mental events is under maximal control of the experimenter (Haxby et al., 2001; Cox and Savoy, 2003; Haynes and Rees, 2005a,b; Kamitani and Tong, 2005; Norman et al., 2006; Haynes et al., 2007; Kay et al., 2008; Miyawaki et al., 2008; Williams et al., 2008; Hassabis et al., 2009; Chadwick et al., 2010; Op de Beeck et al., 2010; Morgan et al., 2011). Advanced methods have been applied for the decoding of perceptual and cognitive attributes. For example, some studies have focused upon patterns of activity distributed over multiple brain regions and related these activity patterns to a semantic model (e.g., Mitchell et al., 2008). As another example, several studies have successfully reconstructed visual pictures and movies from patterns of activity in visual cortex (Kay et al., 2008; Nishimoto et al., 2011).

However, the complexity of everyday mental events goes far beyond the typical application of fMRI-based brain decoding. Such mental events are by definition changing dynamically and involve multiple brain regions and multiple cognitive processes. In order to apply brain decoding methods to such situations the methods have to be adapted to take into account the dynamic and multi-faceted nature of neural processing.

Here we focus in particular upon the specific example of visuospatial navigation. Invasive research in other animals has allowed a detailed characterization of the underlying neural mechanisms at the level of single neurons and population activity. Clearly, the process of navigation as a whole is served by many brain regions (Whitlock et al., 2008; Derdikman and Moser, 2010; Wolbers and Hegarty, 2010; Morgan et al., 2011). The most detailed studies have been performed in rats, in which active navigation was accompanied by selective responses in a wide set of functionally defined neurons, including head direction cells, border cells, and grid cells in parahippocampal cortex, and place cells in hippocampus (Derdikman and Moser, 2010). In addition to these “direct” spatial representations, visuospatial navigation also involves many cortical regions, such as visual, somatosensory, and motor regions. The activity of these regions is indirectly related to spatial processes in the sense that these non-spatial representations convey the information necessary to construct the spatial representations in parahippocampal and hippocampal regions.

In humans, such detailed knowledge is not available. Brain imaging studies, which have focused on virtual navigation given that subjects must remain still during scanning, confirm that multiple brain regions are active during navigation (Gron et al., 2000; Jordan et al., 2004; Rosenbaum et al., 2004; Morgan et al., 2011). Furthermore, these studies have shed some light upon the specific role of each of these brain regions, mostly by using a cognitive subtraction strategy in which the involvement of particular cognitive processes was manipulated by changing task or stimulus attributes during navigation. Specific properties of the functional organization of grid cell representations have also been used to indirectly infer the existence of grid cells in human entorhinal cortex based upon a macroscopic fMRI signal (Doeller et al., 2010).

However, human brain imaging studies have not yet revealed how the content of neural representations in these brain regions relates to what happens when a person is navigating through a (virtual) spatial environment. A few studies have applied brain decoding to retrieve spatial information from neural signals, for example, the quadrant of a virtual room to which a participant has navigated (Hassabis et al., 2009). Based on the signal from hippocampus, the primary area expected to contain a cognitive spatial map (Andersen, 2007), performance in this four-choice decoding problem was close to 50% accuracy (chance performance would be 25%).

Despite the importance of such a success, its limits are immediately obvious and provide the impetus of our current study. First, it was obtained in a very simple environment, and it is known from studies in rats that spatial representations are complicated and hard to make sense of in more complex environments (Crick et al., 2004; Derdikman and Moser, 2010). Thus, given the successful but still limited performance of fMRI-based decoding in a very simple environment, the reliability of reading out the hippocampal spatial map in a more complex environment might be low. So it would seem that decoding someone's location from fMRI data is still very limited, in contrast to the detailed information contained about current and even past spatial position in the responses of single hippocampal neurons (Davidson et al., 2009). Second, only the hippocampus was involved in this fMRI-based decoding, while, as mentioned above, many other brain regions are active during navigation. Visuospatial navigation is a complex event that involves the concerted and dynamically changing activity in multiple brain regions, which is related to the many different motor, sensory, and cognitive processes that are involved in navigation (Wolbers and Hegarty, 2010). Thus, it might be worthwhile to develop a method to use the content of these non-spatial representations in many different cortical regions in order to decode navigation behavior.

Here we provide the first “proof of principle” study demonstrating the potential of brain decoding methods to decode how subjects navigate in a complex environment based on the dynamically changing pattern of activity in multiple brain regions, a method we refer to as “combinatorial brain decoding.” Subjects navigated in a complex virtual environment with many hallways, doorways, and rich visual cues. We illustrate the difficulties faced by traditional decoding methods to decipher spatial location during navigation in a complex maze based on direct spatial information from human hippocampus. Nevertheless, we were able to decode non-spatial information from visual and motor-related processing based on activity patterns in multiple cortical regions. We then adapted our decoding approach to convert this non-spatial information into spatial knowledge taking into account the activity patterns at multiple time points, related to multiple aspects of navigation events, and in multiple brain regions. Using this combinatorial brain decoding approach, we obtained high success in decoding the route that subjects followed through the complex maze.

## Materials and methods

### Procedures

#### Subjects and ethics information

Three subjects participated in the fMRI study, one co-author (S1) and two naive subjects (S2 and S3). Subjects were male, right-handed, and 28 years (S1 and S2) and 25 years (S3) old at the time of testing. One additional subject (male, 23 years old) participated in a behavioral experiment only. The procedures were approved by the Medical Ethical Committee of the KU Leuven and complied with the code of ethics of the world medical association (Declaration of Helsinki). Written informed consent was obtained from each participant.

#### Virtual maze environment

**Figure 1:**
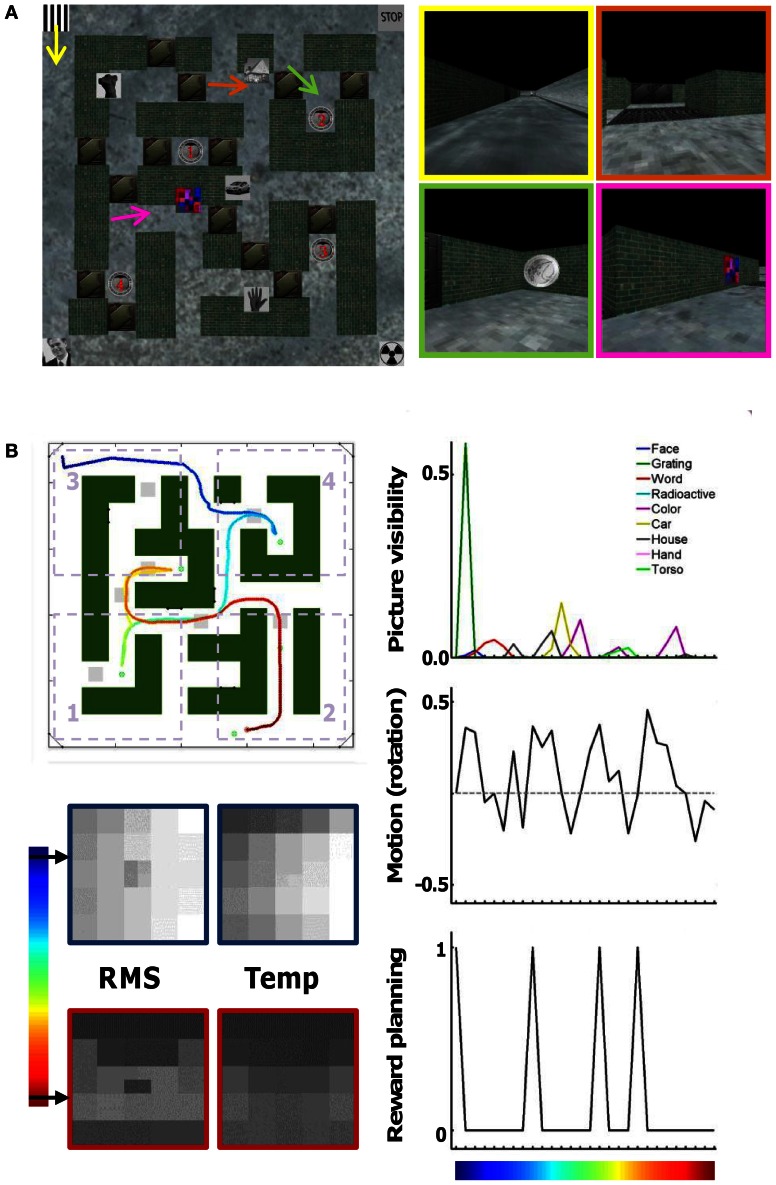
**Virtual maze environment and behavioral parameters. (A)** Map of the maze with the position of brick walls, hallways, doors that could be closed or open, pictures on the wall, and rewards 1–4. On the right are a few snapshots taken at the position and viewing direction indicated by the arrows and with one possible status of the doors. For example, in the top-right screenshot the first door is open but the second door behind is closed. **(B)** Example route (track) taken by a subject. The map of the maze is the same, but now doors are either open (indicated by light-gray squares) or closed (shown in “green,” as are the walls). The color code of the track indicates time (start = “dark blue”). The division of the maze into four quadrants is shown by the dotted rectangles. Other relevant parameters that can identify this track are shown as well: times at which each of the nine pictures is visible and their size, rotational motion (left = positive values), times at which the subject is informed about the next reward to collect, and information about scene content in the spatial domain (differences between pixels, “RMS”) and in the temporal domain (differences across time, “Temp”). These parameters are sub-sampled to the scanning frequency of 1 time point per 3 s (28 time points for this track).

The layout of the virtual maze and first-person screenshots along a particular route are shown in Figure 1A. The virtual maze was constructed using Matlab (MathWorks) and the Virtual Reality Toolbox (MathWorks). We created a prototype textured box that provided the building blocks for our maze. By using this technique, the whole maze was completely controlled through Matlab routines. The maze layout could be read in from an image file (22 by 22 pixels or “tiles”). The position of walls and corridors was kept constant throughout the experiment. There were seven pairs of doors located throughout the maze, and on each trial only one door from each pair was open. The variable status of the doors forced subjects to adapt their planned route throughout a trial. A total of nine pictures (from the internet, some of them obtained through a subscription to photos.com) hung on the walls of the maze at fixed positions. The subjects navigated through the environment by pressing buttons on an MRI compatible button box (Current Designs): three buttons for left, straight on, and right, respectively. The speed of travelling was one tile per 0.78 s. At this speed, travelling the full extent of the long corridors around the outside of the maze would take about 17 s. The “straight on” button had to be pressed continuously in order to move forward (without pressing a button the subject would just stand still), and multiple/long left or right button presses were required for extensive turns. Scenes were refreshed at a rate of 20 frames per s. Supplemental Movie S1 shows the actual maze during the first part of the track shown in Figure 1.

Five rewards were present in the maze. The rewards were in the form of a €2 coin. Four of the rewards were located at fixed positions in the maze. Only one, the current target reward, was visible at any given time. An additional fifth reward was always visible, and could be found at one of four positions along the outer corridor of the maze.

In each trial the order in which the four fixed rewards had to be collected varied. The status of the rewards collected and the current reward goal was indicated below the main window. This area contained five small rectangles numbered 1–5, of which the current target reward was colored yellow, the collected rewards green, and the remaining rewards black. The fifth reward had to be collected before the trial was considered complete, but subjects were free to collect it anytime during the trial.

One hundred and six trials were completed across all scan sessions, each following a different route/track. In the additional behavioral experiment, a control subject completed four re-runs of the 106 fMRI trials performed by S1 and S2. For each re-run the environment and task were exactly the same as during the corresponding scan run: the same doors were open/closed, and the four fixed rewards had to be collected in the same order (again the timing of collecting the fifth reward was up to the subject). This resulted in an additional 284 tracks that differed from those collected during the scan sessions.

Before the start of the experiment, subjects were made familiar with the maze and the task through a practice session. As a result, the duration of trials did not change progressively during scanning. Across all trials obtained during scanning and in the additional behavioral experiment, the trial duration ranged from 57 to 195 s.

During scanning, the stimuli were presented on a Barco RLM 6+ projector using a PC running Windows XP. During behavioral sessions, stimuli were presented on a CRT monitor using a PC running Windows Vista.

### Extraction of behavioral and stimulus parameters during trials

Several variables were extracted from the route followed by subjects on each individual trial. These variables were tested as a source of information to decode a subject's route based on fMRI activity. Each variable was first calculated for each time point in the resolution of the virtual maze software, i.e., 20 frames per s. Afterwards all 60 values falling into the acquisition time (TR) of 3 s for one functional MRI image were averaged to obtain a variable in the same temporal resolution as the fMRI images. These variables are illustrated for one example track in Figure 1B.

#### Quadrant location

The average (*x*, *y*) position of a subject in the maze was calculated per TR, and this average was converted into a four-value variable indicating in which quadrant a subject was situated at each point in time. Figure 1B shows the boundaries of the four quadrants. Positions in the middle were not assigned to a quadrant and these time points were not used to train the decoding classifiers.

#### Picture visibility

This variable was calculated for each of the nine pictures separately. The score was equal to the visible area of the picture when the image was at least partially visible. A non-visible picture would have value zero. A maximal value of one would mean that the picture would fill the total scene (nothing else visible).

#### Motion path

Rotational motion from one frame to the other. This variable can have negative values (turning right), zero values (stationary or moving straight ahead), or positive values (turning left).

#### Timing of reward planning

This variable is one for the *TR* when a subject first received information on which reward to collect next, and zero otherwise.

#### Stationary scene content (scene RMS)

Each scene was divided into a grid of 5 × 5 squares. The middle square was further divided into four smaller squares, yielding 28 values per scene. For each square and each scene/time point we summarized the content of each square region by the standard deviation of the luminance (gray-level) across the scene pixels in the area of the square.

#### Temporal scene content (scene Temp)

The same 28 squares were used. Per pixel in a square we calculated the difference in gray-scale value between two frames, we only retained the positive values (increase in gray-scale values are most easily interpreted as onset of stimuli), and summed these positive values over all pixels in a square.

We decided to use these summaries of scene content in terms of a small subset of squares to keep the decoding task tractable (each of the squares is in fact a separate variable on which a classifier was trained). As a consequence, scene RMS and scene Temp only contain a very crude representation of scene content, and most scene information is not used. Nevertheless, given the relatively poor spatial resolution of fMRI, we hoped we would retain these aspects of the scene content that would dominate the pattern of fMRI activity and that is most responsible for decoding natural image content as shown in a few recent studies (Kay et al., 2008; Miyawaki et al., 2008; Nishimoto et al., 2011). While these studies were merely using this information to classify the scenes itself, we are going a step further and infer spatial location from it.

Note that the scenes, and our summarized version of it, are all very similar to each other, much more than most random pairs of natural images. First, most scenes have a similar layout, with e.g., a triangular darker region on top indicating the sky surrounded by two walls (see Figure 1B). After reduction to the 28-square format, little information seems available to decide where a subject is or what he/she is looking at. Second, scene content was determined without taking eye movements into account, and any eye movements would be expected to degrade decoding based on the activity pattern in a retinotopic map (see Section Eye movements during navigation).

### Eye movements during navigation

During the first scan session subjects were free to make eye movements (there was no instruction to fixate, nor was a fixation cross present). After this session subjects were instructed to fixate, and several training trials with a fixation point present were completed outside the scanner. For two of the subjects, subjects (S1 and S2), we recorded eye movements during a period of 6 min of navigation with an EyeLink I infrared eye tracker. The eye fixation data revealed that subjects mostly fixated straight ahead when moving forward, which is the most prevalent direction of motion. When moving left or right, they made horizontal saccadic and pursuit eye movements. Some saccades were also made to the bottom of the screen where information was given about which reward to collect next, which usually occurred around the time of finding the previous reward. Overall subjects fixated in the central two visual degrees for 11% (subject S1) and 40% (S2) of the time.

In the second scan session, a fixation cross was present and all three subjects were instructed to fixate, which avoided most of the horizontal eye movements when turning. Testing with an eye tracker prior to this scan session showed that subjects were able to fixate in the central two visual degrees for 90% (S1) and 66% (S2) of the time.

For the decoding of people's whereabouts we partially rely on visual information (the “scene content” cue). This information was determined without taking eye movements into account, and any eye movements would be expected to degrade decoding based on the activity pattern in a retinotopic map. Nevertheless, the decoding results show that scene content information was extremely useful for the decoding of a subject's location for both scan session 1 (in which subjects were freely viewing) and scan session 2 (in which subjects were requested to fixate centrally), without an obvious difference between the two scan sessions in the decoding based upon this variable. This indicates that the variation in eye position during navigation as reported above does not have a strong influence on fMRI decoding based on patterns of activity in visual cortex.

### Scanning parameters

A Siemens 3 T Magnetom Trio MRI scanner (Siemens, Erlangen, Germany) with an eight-channel head coil was used for image acquisition. For both subjects, a high resolution T1-weighted structural image was acquired using a magnetization prepared rapid gradient echo sequence [MPRAGE; *TR* = 2300 ms, echo time (TE) = 2.98 ms, 1 × 1 × 1.1 mm voxels, field of view (FOV): 240 × 256, 160 sagittal slices]. Functional data (fMRI) were acquired with a descending gradient EPI pulse sequence for T2^*^-weighted images (*TR* = 3000 ms, *TE* = 30 ms, flip angle = 90°, 50 oblique axial slices each 2.8 mm thick, inter-slice gap 0.028 mm, in-plane resolution 2.5 × 2.5 mm, 80 × 80 matrix).

### fMRI analyses

#### Pre-processing

The Statistical Parametric Map software package (SPM5, Wellcome Department of Cognitive Neurology, London) was used for pre-processing fMRI data, which involved correction for timing of slice acquisition, realignment (correction for motion; in two steps with registration to the mean and 3rd-degree B-spline interpolation), coregistration of anatomy to the mean functional image, segmentation and spatial normalization of the anatomy to an MNI (Montreal Neurological Institute) template, normalization of the functional images (images resliced to a resolution of 2.5 mm isotropic), and spatial smoothing of the functional images with a Gaussian kernel of 5 mm full-width-at-half-maximum.

#### Statistical whole-brain analysis

**Figure 2:**
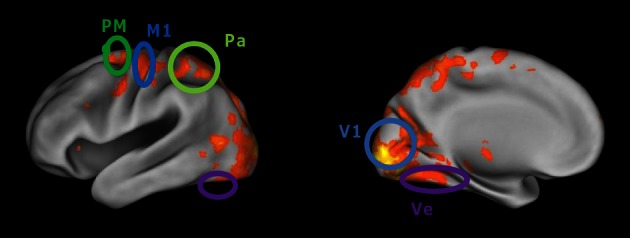
**Cortical regions activated more during virtual maze navigation than during resting blocks.** Activity was thresholded at *p* < 0.0001 (corrected for multiple comparisons). The cortical regions of interest defined in our analyses are circled: primary visual cortex (V1), ventral visual cortex (Ve), superior parietal cortex (Pa), primary motor cortex (M1), and dorsal premotor cortex (PM).

The data from the first session of each subject were modeled with a general linear model applied to pre-processed images, with one independent variable indicating the time and duration of navigation blocks and six covariates (the translation and rotation parameters derived from re-alignment). To find voxels activated by navigation, the contrast of navigation vs. rest (fixation blocks) was calculated and thresholded at *p* = 0.00001, family-wise corrected for multiple comparisons and only included clusters above 40 voxels. An example activation map is shown in Figure 2.

The hippocampus was not activated in this contrast in any of the subjects, even at much more lenient thresholds (e.g., *p* = 0.05, uncorrected). We implemented some expanded general linear models with specific variables chosen to activate hippocampus. We hypothesized that this area, directly involved in the representation of a spatial map of the environment, might not be active during the whole navigation period but only at times when a new route has to be planned. We expected such route planning to be most prominent at the start of each trial and when the identity of the next reward is revealed, which is also the moment at which the previous reward has been collected. The inclusion of an independent variable indicating these moments still did not reveal any activated voxels in hippocampus (uncorrected threshold of *p* = 0.001). It is possible that hippocampal activation would have been found with other functional MRI sequences, e.g., with a higher spatial resolution. In addition, several studies in the literature have indicated that a resting period is not a good contrast condition to find differential hippocampal activation, because hippocampus is part of the default mode network which is typically active during rest breaks (Greicius et al., 2004).

#### Selection of regions of interest

The cortical regions of interest (ROIs) are defined based on a conjunction of anatomical and functional criteria. *Primary visual cortex (V1)* was defined anatomically by a mask based on a probabilistic map of area 17/V1 in MNI space, derived from the Anatomy toolbox for SPM (www.fz-juelich.de/ime/spm_anatomy_toolbox). In this anatomical region, we included all voxels that were activated by navigation (using the contrast and threshold mentioned above; subject S1: 1561 voxels, S2: 953 voxels, S3: 669 voxels). *Ventral visual cortex (Ve)* was determined anatomically at the ventral surface of the occipital and temporal lobes. We included all voxels in this region activated by navigation (S1: 1101 voxels; S2: 840 voxels; S3: 330 voxels). The *parietal (Pa)* ROI was located in the more anterior and superior parts of the parietal lobe (S1: 515 voxels; S2: 607 voxels; S3: 563). The anatomical definition of ventral and parietal cortex was conservative in the sense that these ROIs only included a part of the region that would normally be considered for inclusion, namely the part furthest removed from area V1. This definition assures that these regions and V1 were clearly separated from one another. The definition of *primary motor cortex M1* (S1: 114 voxels; S2: 171 voxels; S3: 122 voxels) and *dorsal premotor area PMd* (S1: 221 voxels; S2: 349 voxels; S3: 254 voxels) was also based on their typical location and activation in the contrast of navigation vs. rest epochs. We selected these five brain regions because they represent the whole extent of activity which we see when comparing the navigation periods with the baseline, and because from the literature we can expect them to contain sensory, motor, or cognitive representations which are at least indirectly implicated during visuospatial navigation.

*Hippocampus (Hi)* was determined solely on anatomical criteria due to the absence of any navigation-related activation (S1: 384 voxels; S2: 273 voxels; S3: 635 voxels). Importantly, inspection of the EPI images and the extent of susceptibility artifacts suggested that there was sufficient signal around the location of the hippocampus.

Finally, a *white-matter* ROI was selected around the corpus callosum as a control region (S1: 935 voxels; S2: 674 voxels; S3: 830 voxels).

#### Extraction of fMRI signal in ROIs

For each voxel in each ROI, we selected the fMRI signal in the pre-processed images for each time point, i.e., a resolution of 1 time point every 3 s. This raw signal was normalized for any differences between runs by subtracting the mean fMRI signal across all ROI voxels across all time points of each run. Furthermore, the signal was normalized for any linear and quadratic drifts across each run. To account for the typical delay in the hemodynamic response function, the fMRI signal was taken from two *TR*s (6 s) after each point in time.

### Decoding of maze behaviour and tracks from fMRI signals

#### General training and cross-validation procedure of support vector machine (SVM) classifiers

Linear support vector machine (SVM) was implemented using the OSU SVM Matlab toolbox (www.sourceforge.net/projects/svm/), with its default parameters (e.g., parameter *C* = 1). We trained a classifier using all time points from all trials minus one, and tested it on the individual time points of the remaining trial. This procedure was repeated so that each trial was tested. The time points of the training trials were used for training the classifier to find a hyper plane in the multidimensional voxel space that best separates the voxel response vectors of the two categories that the classifier is required to separate. All time points of the one remaining trial were then used to test the classifier. In particular, the classifier's output was used to determine the probability of which category occurred at each time point of the trial (see further paragraphs on *SVM output* and *Choosing a track*).

We included a separate training/test cycle (separate decoding) for each source of information. These sources of information were calculated as specified before (see Section Eye movements during navigation), but in addition each source was transformed into a binary variable (e.g., the motion path variable was transformed into “turning left” vs. “turning right”). Furthermore, for most sources of information only a subset of time points from the training trials were used to train the classifier. For the *quadrant location*, the selection was already made during the calculation of the variable (positions at the boundaries between quadrants were not used). For the *motion path*, which in principle combines different information (turning vs. not turning and turning left vs. turning right) the training time points were restricted to the time points with the lower third (negative values, turning right) and the upper third (positive values, turning left) of the values. This way the classifier is mostly trained on discriminating the direction of turning. For *reward timing* and *picture visibility*, only a subset of the no-reward and the no-visible trials were used to avoid a larger than 3–1 relative frequency of “no” to “yes” time points during training. Finally, for *scene content* all values in a square of the scene were used, and the categories were determined as below or above the median value of all values in that square across all training trials. All these choices were made a priori, and none of them was changed a posteriori to improve decoding performance. This avoids any statistical bias in our findings, but it also means that decoding performance can probably still be improved significantly by systematically manipulating these factors and finding the best combination of values.

**Figure 3:**
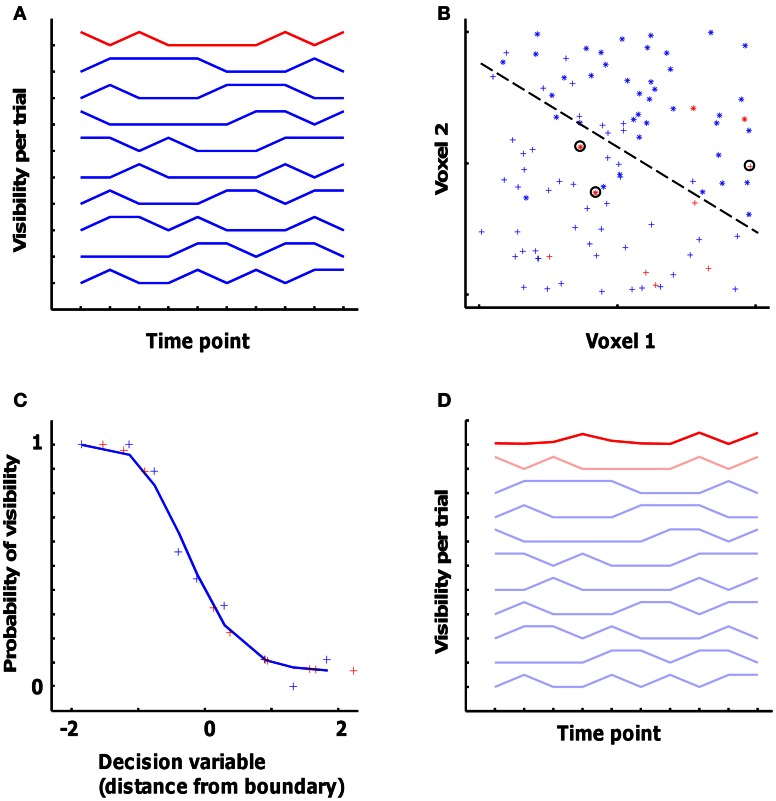
**Simulation dataset to illustrate the pattern classification approach applied to the fMRI data. (A)** Visibility (0 or 1) for 10 ‘trials’. Nine trials, shown in blue, are used for training the classifier, with one remaining trial (shown in red) left out for cross-validation. **(B)** The fMRI signal in two simulated voxels at each of the 10 × 10 data points. The 9 × 10 blue data points are involved in training together with the associated visibility label (represented by the ‘+’ and ‘^*^’ symbols). The dashed line represents the optimal decision boundary to separate the visible from the non-visible category. The 10 red data points represent the cross-validation data, 3 out of which are classified wrongly (encircled points). **(C)** The probability of visibility in the training data as a function of the decision variable of the classifier. The blue plus-signs represent the data from the training dataset (binned so that each point is an average of 9 data points), and the red plus-signs represent the cross-validation data. The blue curve shows a sigmoid function fitted to the training data. **(D)** The actual visibility in the 9 training trials and in the cross-validation trial from panel **(A)** complemented with the predicted visibility (bold red line) in the cross-validation trial as obtained from the voxel-based classifier.

Figure 3 uses simulated data to illustrate SVM training. Figure 3A shows the visibility (in this case, 0 or 1) for 10 “trials” (the individual line plots), each with 10 time points (time is on the *X*-axis). Nine trials, shown in blue, are used for training the classifier, with one remaining trial (shown in red) left out from training and used to cross-validate the classifier. Figure 3B shows the fMRI signal in two simulated voxels at each of the 10 × 10 time points. For each of the 9 × 10 blue time points we have a vector in the two-dimensional voxel space and the associated visibility label (represented by the “+” and “*” symbols), and these (vector, label) pairs are used for training the classifier to find the optimal decision boundary to separate the visible from the non-visible category.

For cross-validating the classifier with the fMRI data from the test trial, data from each time point in the test trial are placed in the voxel space (red points in Figure 3B). Two parameters describing the relationship of this point to the decision boundary are relevant for what is done next. The first parameter relates to the side of the boundary the point falls in. This determines the category that is chosen by the classifier, i.e., whether it is predicted to be a “visible” or a “non-visible” time point. In this case, the classifier made a correct decision for 7 out of 10 time points in the test trial (the three errors are circled). The second parameter is the distance of the point in space from the decision boundary. This distance can be seen as the certainty of the classifier for classifying this time point as belonging to one or the other category.

Note that many classifiers were trained for each trial. First, separate classifiers are trained for each ROI. Second, there were six sources of information, as specified above. Some of these information sources were themselves decomposed into multiple variables: quadrant location provides six possible pairs of quadrants, picture visibility has nine variables (one per picture), and stationary and temporal scene content each contain 28 variables. A classifier was trained for each variable. Summing across all sources of information (see Section Extraction of behavioral and stimulus parameters during trials, for the list) and across all variables per source, there is a total of 73 classifiers per ROI per trial.

#### SVM output: binary classification vs. predicted probability

SVM output is typically expressed as a binary classification. Successful decoding is reflected by a higher than chance classification of the individual time points of the trial used in the cross-validation test. In a two-choice situation with equal occurrence of both alternatives, expected chance performance is 50%. However, we often have an unequal occurrence of both alternatives, and then the expected chance performance is higher. To control for this, we calculated the proportion of hits (e.g., the classifier responding “visible” when a picture was indeed visible) and false alarms (e.g., the classifier responding “visible” when a picture was not visible), and we derived the performance that would be achievable with the difference between hits and false alarms if both alternatives would have an equal probability of occurring. Take, for example, a classifier that reaches 90% correct by simply classifying all input patterns into the response alternative that would be correct for 90% of the patterns. In this case, the actual accuracy, obtained without extracting any information from the input patterns, would be 90%, but the corrected accuracy would be 50% correct. These are the accuracies mentioned in this text, so for all of these values 50% is the expected chance performance when the input patterns do not convey any useful information for the classification problem. To calculate this corrected accuracy, we first calculated the psychophysical measure of sensitivity (**d′**) as follows:

$$d^{\prime }=z(H)-z(F)$$

(with **H** the proportion of hits, **F** the proportion false alarms, and *z* the inverse of the normal cumulative distribution.)

And we derived the corrected proportion correct as follows:

$$P=normcdf({\text{d}}^{\prime }/2)$$

(with *normcdf* the value of the normal cumulative distribution)

As mentioned above, the classifier bases its decision on the position of a data point in the input voxel space. There is more to this position than the category (side of the decision boundary) it falls in. The classifier also informs us about the certainty of this decision, which is a more graded and more informative measure than the binary classification. This certainty is reflected by the distance between a data point and the decision boundary. We related this distance and its sign (side of the decision boundary) to the expected value of the input data by fitting a function to the observed relationship between the value of input data points and the decision and certainty of the classifier.

This is illustrated further in Figure 3. In Figure 3C, the *X*-axis shows different values of the “decision variable” (DV) computed by the classifier, which is the signed (negative or positive) distance from the decision boundary [these data are binned: each point is a mean of 9 time points; the leftmost point is the average (*x*, *y*) of the 9 time points with the most negative DV; in the actual data, averages of 20 time points were used for binning]. The *Y-axis* shows the mean probability of the visibility for each of the mean DV values shown on the *X*-axis. The blue plus-signs are the data from the training dataset, and the red plus-signs are from the remaining trial used for cross-validation. This plot illustrates how different values of the DV relate to the probability of picture visibility. In order to use this relationship for all possible values that might be encountered in the test data, we need to obtain a continuous function. To do this, the (*x*, *y*) pairs were fitted with a sigmoid of the type

y=f(x)=c+(1.0−c−d)/(1+exp((x−a)/b))

(a, b, c, and d are the fitted parameters)

The decision to use this fitting procedure and this function with these parameters was made after visual inspection of the typical relationship between the DV and the probability of picture visibility in the first subject. There are other mathematical approaches to characterize this relationship between the DV and probability, such as regularized linear discriminant analysis and regularized logistic regression.

This sigmoid function is approximated by the blue line in Figure 3C. This function is fit in each cross-validation fold using the training trials. This same function was then used to link the observed DV values for the test trial to a predicted value of the input variable, e.g. picture visibility (red plus signs in Figure 3C). Another format to display these predicted values, is with the 10-point time line in Figure 3D. The dark red line connects the same 10 predicted values, but now in order of time. A comparison of the dark red (predicted values) and light red (actual values) lines reveals that the prediction is clearly incorrect for three time points (time points 1, 3, and 4), which are the data points that were incorrectly classified (circled crosses in Figure 3B).

#### Choosing a track: comparison of predicted values with actual information

The decision of which track a subject followed in a specific trial was based on a comparison of the predicted values calculated by a classifier with the actual values encountered in all available tracks. In Figure 3D, this would be a comparison of the dark red line with the other 10 lines.

The metric used to compare the predicted values with the actual values encountered in each track was a sum of squares measure (SS_diff) of the difference between predicted and actual values. If the information source included multiple variables, such as picture visibility (which included nine variables; one for each picture), then all variables and their predicted values were concatenated to yield two long vectors of predicted vs. actual values. To calculate SS_diff, each pair of predicted and actual values was first re-sampled by linear interpolation to have the same number of time points across all tracks (the number of samples was set to the number of time points contained in the longest track), and standardized separately to a mean of 0 and a variance of 1. Then, the difference between the predicted and actual values was calculated per time point, this difference was squared, and then summed across all time points to obtain SS_diff. Finally, all available tracks were ranked according to this SS_diff, with the smallest SS_diff given rank 1. The track with rank 1 is the track that the classifier output picks as the best possible guess of the actual track followed during the trial.

One way to summarize the decoding performance is to determine the number of trials in which the correct track comes out on rank 1. Another approach is to investigate the mean rank of the correct track across all trials. With random data, and as verified by our control data (see further), the expected rank is

rank(random)=(nrTrials+1)/2

(nrTrials equals the number of alternative tracks. With data containing reliable information, this correct-track rank will be lower).

The rank expected by chance depends on the number of alternative tracks. We started with the pool of tracks/trials performed in single scan sessions, giving a total number ranging from 16 to 19. In further analyses, we worked with a pool of all 106 trials completed during the scans, and we determined the rank of the correct track for each of the 106 trials. Finally, we extended the pool to include the 284 additional trials completed outside the scanner by subject VS, and in this case we determined the rank of the correct track, which could now range between 1 and 390, for each of the 106 fMRI trials.

The dependence between expected rank and number of alternative tracks can be taken into account by dividing the correct-track rank by the total number of trials plus 1. This “normalized correct-track rank” is expected to be 0.5 in a case in which decoding fails. It will be one divided by the number of alternative tracks if the correct track is chosen on every trial (100% accuracy). The data show that performance is relatively invariant to the number of tracks when expressed with this normalized correct-track rank (e.g., the high similarity of the 106-alternatives and the 390-alternatives panel in Figure 5). Thus, we can use the performance of our methods with a particular number of alternatives to predict how accuracy will scale with much higher numbers.

### Statistical notes

#### Threshold for significance

**Figure 4:**
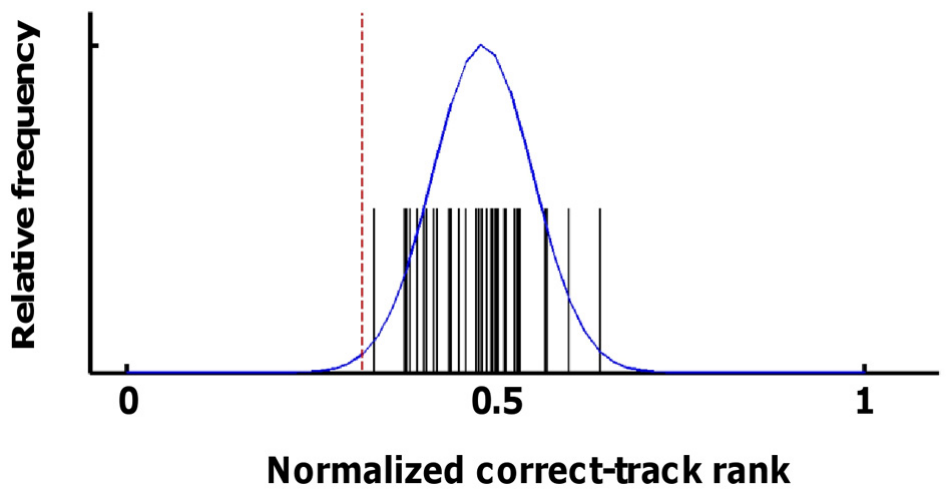
**Statistical distribution of correct-track ranks in the absence of signal.** Here we show the distribution of the correct-track ranks observed when applying pattern classification on the data from the white-matter ROI using the various sources of information and ROIs that are shown in Figure 6. The black spikes represent the individual values, the blue line is a normal distribution fitted to these data. The red line corresponds to the statistical threshold used for determining whether decoding performance was better than chance.

The distribution of correct-track ranks that is expected by chance has an expected mean equal to the number of trials plus 1 divided by two, resulting in a normalized correct-track rank of 0.5. A critical test that this assumption holds for our data was provided by a white-matter ROI. Classifiers were trained and tested using input data from these white-matter voxels (separately for the three white-matter ROIs of the three subjects), and with all the sources of information and pools of alternative tracks (individual sessions or larger pools) shown in the Results section. The obtained values and a normal distribution fitted to these values are shown in Figure 4. Across all 40 values, the normalized correct-track rank had a mean of 0.482 and a standard deviation of 0.068. We considered decoding to be significant if the normalized correct-track rank fell below 2.4 times the standard deviation (normalized rank ≤ 0.32), which corresponds to a significance level of 0.01 when assuming a normal distribution.

**Figure 5:**
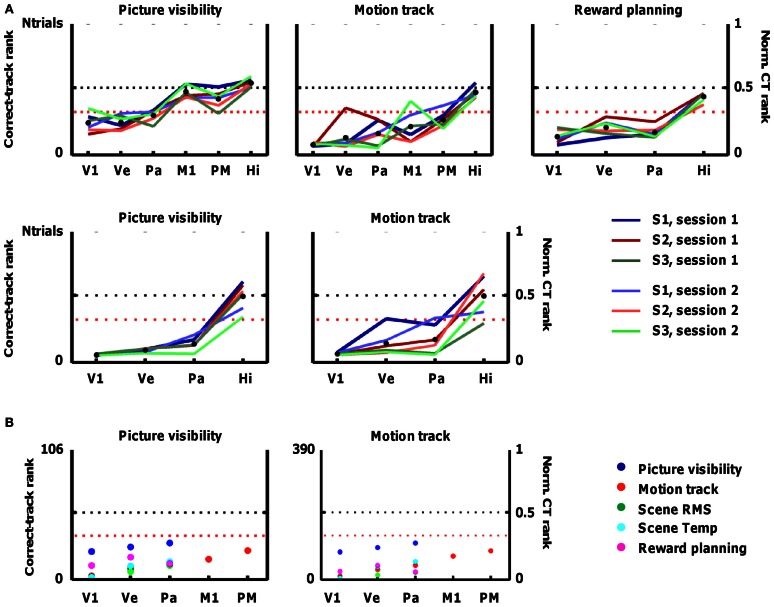
**Accuracy of fMRI decoding in predicting the track that subjects followed in each trial.** The fMRI data are used to predict the occurrence of particular sources of information (e.g., picture visibility) at every time point, and the alternative tracks are ranked based on how much they resemble this prediction across all time points. Accuracy is represented by the correct-track rank (left *Y*-axis), which is the rank given to the correct track when all alternative tracks are ranked according to how much they deviate from the prediction. The right *Y*-axis shows the normalized correct-track (CT) rank, which is the correct-track rank divided by the number of alternative tracks plus 1. A normalized correct-track rank of 0.5 corresponds to chance performance (no decoding ability; black dotted line). Values below the red dotted line represent decoding performance that is significantly better than chance. Data are included for the following regions of interest: primary visual cortex (V1), ventral visual cortex (Ve), superior parietal cortex (Pa), primary motor cortex (M1), dorsal premotor cortex (PM), and hippocampus (Hi). **(A)** Decoding performance when the correct track is ranked among all tracks completed in a single scan session (18 on average). The different lines represent the different scan sessions. **(B)** Decoding performance when the correct track is ranked among all 106 tracks completed in the fMRI sessions (left panel), or among all 390 tracks completed overall (right panel). The different colors represent the different sources of information.

This threshold is not corrected for multiple comparisons and is not sufficiently conservative for assessing significance for each combination of sources and ROIs in each participant. Thus, for statistics, we additionally relied upon consistency across all six scan sessions. With random data, the probability of observing that a particular combination of source and ROI is on average below the same threshold for significance is much lower than the 0.01 probability of observing this value in a single scan session. To assess this probability of on average crossing the threshold given random data, we used the distribution of 40 values obtained with the white-matter ROIs; We randomly selected six out of these 40 values and computed the mean of these six values; we performed this procedure 5000 times and we calculated the mean and standard deviation of this “mean of 6 values” distribution. This distribution had a mean 0.48 and a standard deviation of 0.027. The threshold of 0.32 is 5.97 times the *SD* lower than this mean, corresponding to an (uncorrected) probability of *p* < 10^−8.^ Even a conservative Bonferroni correction for 100 multiple comparisons (which is much more than the actual number) results in a corrected significance level of *p* < 10^−6^. In Figure 5A, 17 combinations of information sources and ROIs demonstrated a mean (across all six scan sessions) correct-track rank below this threshold. These 17 combinations were later used to test for the effect of integrating across sources and ROIs.

In further analyses we tested decoding performance by selecting the correct track from a larger pool of alternative tracks, namely all fMRI trials and even trials completed outside the scanner. In these analyses, effects of combining ROIs and sources of information (Section Combinatorial brain decoding: combining predictions across ROIs and across sources of information and Table 1), or effects of similarity (Section Specific benefits of combining ROIs and/or sources of information) were tested statistically by determining how consistent the effects were across the 106 fMRI trials.

**Table 1:** **Correct-track ranks obtained when combining the predictions of ROIs and sources of information**.

**Combined sources/ROIs**	**Rank**	***SEM*^1^**	**Best single rank**	**Control rank^2^**
1. All sources, all rois (*N* = 17)	1.07	0.03	1.97	165.46
2. Only visibility, all 3 ROIs	66.58	8.17	80.38	187.79
3. Only motion, all 5 ROIs	11.40	2.24	10.68	200.81
4. Only RMS, all 3 ROIs	3.82	0.69	1.97	131.22
5. Only Temp, all 3 ROIs	13.21	2.57	37.79	168.81
6. Only Reward, all 3 ROIs	12.51	2.38	22.82	183.13
7. Only V1, all sources	1.31	0.10	1.97	165.46
8. Only ventral, all sources	5.58	1.18	13.50	165.46
9. Only parietal, all sources	6.64	1.55	22.13	165.46
10. Everything except V1	1.48	0.13	13.50	165.46

1 For each of 106 fMRI trials the correct track is selected from a pool of 390 tracks, SEM is the standard error of the mean across the 106 fMRI trials.

2 “Control rank” is the rank obtained with the white-matter control region when using the same sources as in the other columns.

#### Statistical bias

Another matter of importance in fMRI decoding methods is the possibility of statistical bias in the analyses. In general, it is advisable that ROIs are selected based on criteria that are statistically independent of within-trial information and between-trial consistency (because the latter is what matters for classifier performance). In our case the ROIs are selected based on the contrast of navigation vs. rest in the first scan session. This contrast does not rely on within-trial information and between-trial consistency of this within-trial information. A further assurance of any statistical bias is that the source × ROI combinations that resulted in better than chance performance in the first scan session, also performed well (and overall at least as good) in the second scan session (as mentioned in the previous paragraph), even though the data from the second session were not used to determine which voxels were activated.

A more subtle statistical bias might arise when we combine ranks across source × ROI combinations (see Results section). In this procedure, we weighted each source × ROI according to its decoding performance by subtracting its normalized correct track-rank from 0.5.

In this case, the data used to determine this weight are not independent from the data that we use to test the effect of integrating ranks across sources and ROIs. We performed two tests to make sure that the beneficial effect of integrating ranks is not due to statistical bias. First, we applied the procedure of integrating ranks to the more limited problem of finding the correct track among all tracks completed in the same scan session (so with a small pool of alternative tracks). We compared the effect of integrating ranks between two situations. In the first situation the weights were determined based on decoding performance in the same session as the data used to test the effect (but note that the decoding performance itself is still based on a cross-validation with independent data!). In the second situation the weight selection was based on data from a different session than the data used to test the effect. The beneficial effect of integrating information was equally large in the two situations. This is actually not surprising given the finding described in the previous paragraph that decoding performance was very highly correlated between sessions.

A second test was based on the decoding performance of the white-matter ROI in the 355-alternatives problem. This ROI was trained with each source of information, and the average normalized correct-track rank based on a single source of information was 0.45. Then we integrated the ranks across sources, using the correct-track rank to weight each rank, exactly as we do for the source × ROI combinations with successful decoding. The average rank of the correct trial was 170, corresponding to a normalized correct-track rank of 0.43, which is below 0.50. Thus, our procedure for determining the weight given to sources and ROIs when integrating information does not introduce a statistical bias.

## Results

We first demonstrate the limits of typical decoding methods to decipher spatial location in more complex mazes, and we implement a new decoding approach that is not subject to these restrictions. In our study, subjects navigated in a complex virtual maze (Figure 1A). This environment was very rich in terms of potential routes or tracks, changing from trial to trial because of the presence of doors that could be open or closed, and in terms of visual input—including the presence of pictures on the walls showing stimuli such as a face, hand, or car. On each trial, subjects started at an outer side of the maze, and were asked to collect five rewards, of which four were in a fixed location and had to be collected in a specified order. The path taken by a subject on a representative trial is shown in Figure 1B. Three subjects (S1–S3) completed a total of 106 trials (average of 18 trials per session for each subject).

### Limits in decoding spatial location from activity patterns in hippocampus

We first implemented a traditional decoding approach, and we attempted to decode in which of two quadrants of the maze the subject was at every single time point based on the pattern of activity in the hippocampus. We trained a SVM classifier on pair-wise quadrant discriminations using the fMRI data from all first-session trials but one, and we cross-validated the classifier on the classification of the individual time points in the remaining trial (this leave-one-trial-out procedure was repeated for each trial, and for each quadrant pair). The equal-bias (assuming equal number of time points in each quadrant, so that chance performance is 50%) classification performance on this two-choice task was not significantly better than 50% (S1: 52 ± 2%; S2: 54 ± 2%; S3: 50 ± 2% correct; error term represent the standard error across trials). So even on this admittedly very simple question, the traditional decoding approach largely fails in a complex maze. Given the low number of subjects in our study it is possible that a larger study with more subjects might still find an effect that reaches significance in decoding the same two-choice task in this complex maze environment, but we can safely conclude from this first test that the absolute performance level will not be high. Thus, decoding spatial position in a complex maze from hippocampal patterns of activity is unreliable at best at the spatial resolution and field strength used here.

### Decoding indirect spatial information: picture visibility and ventral visual cortex

Many distributed brain areas are activated during visuospatial navigation in our virtual maze (Figure 2). Only a few areas, such as the hippocampus and the entorhinal cortex, are known to contain direct navigational information in the form of a cognitive map. However, place cells in hippocampus and grid cells in entorhinal cortex have access to multiple sources of information to “know” where an animal is in the environment, including visual information (such as the position of environmental landmarks) and motor information (how far and in what direction did the subject move). Our approach in the remainder of this text will be to mimic what these cognitive map neurons do: Decode a subject's track and hence position in the maze from the available visual and motor information in the activated cortical areas.

To illustrate our approach, consider as a potential source of information the nine pictures within the maze, each changing in visibility and size when the subject moves (Figure 1B). Ventral visual cortex, part of the object-vision pathway, is expected to be a primary brain region carrying information about whether specific pictures are visible or not. We trained and cross-validated SVM classifiers on decoding picture visibility (visible or not at each time point) from brain activity patterns in this ROI, taking advantage of the modular organization of the ventral visual cortex where e.g., faces activate an anatomically distinct area compared to houses. Classification performance on this two-choice task was indeed significantly higher than 50% (S1: 61 ± 1%; S2: 67 ± 2%; S3: 60 ± 2%; *p* < 0.01 in each subject according to a *t*-test across trials as well as according to a permutation analysis with permuted labels), indicating that the pattern of activity in ventral visual cortex is informative about the visibility of individual pictures.

### From picture visibility to spatial position and route information

fMRI decoding studies mostly stop here, at the point of classifying brain activity at individual time points or averaged across time points based on the pattern of selectivity in a specific ROI. However, here we extend this typical decoding approach in several ways to select which route or “track” was followed by a subject from the 18 tracks completed in a typical fMRI session. The procedure is explained in detail in methods Section Decoding of maze behavior and tracks from fMRI signals. First, for the test track (on which the classifier was not trained) we extracted an un-thresholded, predicted probability of picture visibility from the classifier to estimate how likely it is that a picture was visible or not given the observed pattern of fMRI selectivity in the test track. Second, we used a sum of squares difference measure for comparing the predicted probability across all time points and all pictures of the test track to the actual visibility in all possible tracks, the test track included. All tracks were ranked, with the track with the least difference between predicted probability and actual visibility on rank 1. Ideally, the correct track would be given rank 1.

Based on picture visibility decoded from ventral visual cortex, the average rank of the correct track was significantly closer to 1 (S1: 4.2; S2: 3.9; S3: 5.24) than expected by chance (expected rank: 9.5; see Section Statistical notes for explanation of how significance was determined). Significant decoding of the followed track based on picture visibility was also found based on primary visual cortex (V1), and parietal cortex, but not based on motor regions, hippocampus, or a white-matter control region (Figure 5A; first panel). Thus, the decoded probability of picture visibility based on activity patterns in visual regions is a useful source of information to decide which track subjects followed in a particular period of time.

### Decoding routes from other sources of non-spatial information

Other sources of information might also be related to patterns of activity in these cortical regions (see Figure 1B). Apart from picture visibility, we trained separate classifiers on (1) the motion path followed by subjects (the degree of turning left or right at specific time points), (2) the times at which subjects learned about which reward to find next, (3) a measure of the local spatial contrast in the visual scenes (scene RMS, which reflects the difference between nearby pixels in each scene), and (4) a measure of the local temporal contrast in the visual scenes (scene Temp, which reflects how much pixel content changes across time). All of these sources of information elicited reliable patterns of selectivity in V1, ventral, and parietal cortex that were useful for classifiers, and the motion path was also useful in combination with activity in motor regions (Figure 5A). In some cases, identifying the correct track was very successful. In the best combination, activity patterns in V1 associated with scene RMS information, almost all tracks in a session were assigned correctly as the average correct-track rank was close to 1 (S1 session 1: 1; S1 session 2: 1; S2 session 1: 1.05; S2 session 2: 1.11; S3 session 1: 1; S3 session 2: 1).

In Figure 5A, 17 combinations of information sources and ROIs demonstrated a mean (across all six scan sessions) correct-track rank below the threshold of significance (see Section Threshold for significance).

The between-session replicability of the decoding performance of particular source × ROI combinations was very high. Specifically, the correlation between the correct-track rank in the first session and the correct-track rank in the second session across all source × ROI combinations shown in Figure 5 (*N* = 24; a few other combinations and other ROIs, e.g., including basal ganglia and Brodmann area 45, were explored but were always at chance) was very high in each subject (S1: *r* = 0.88; S2: *r* = 0.89; S3: *r* = 0.88). This observation provides an internal replication within our study. The correlations in correct-track rank across source × ROI combinations were also very high (always > 0.8) when comparing sessions of different subjects, thus we find essentially the same pattern of decoding performance in the three subjects.

### Decoding ability with a larger space of alternative routes

We further tested the robustness of our method by increasing the number of possible tracks to all trials completed in fMRI over all scan sessions, i.e., 106, or even to a larger pool of 390 tracks. Performance remained high, particularly for scene RMS and the V1 ROI, with a mean correct-track rank of 1.97 with 390 alternative tracks to choose from (Figure 5B).

**Figure 6:**
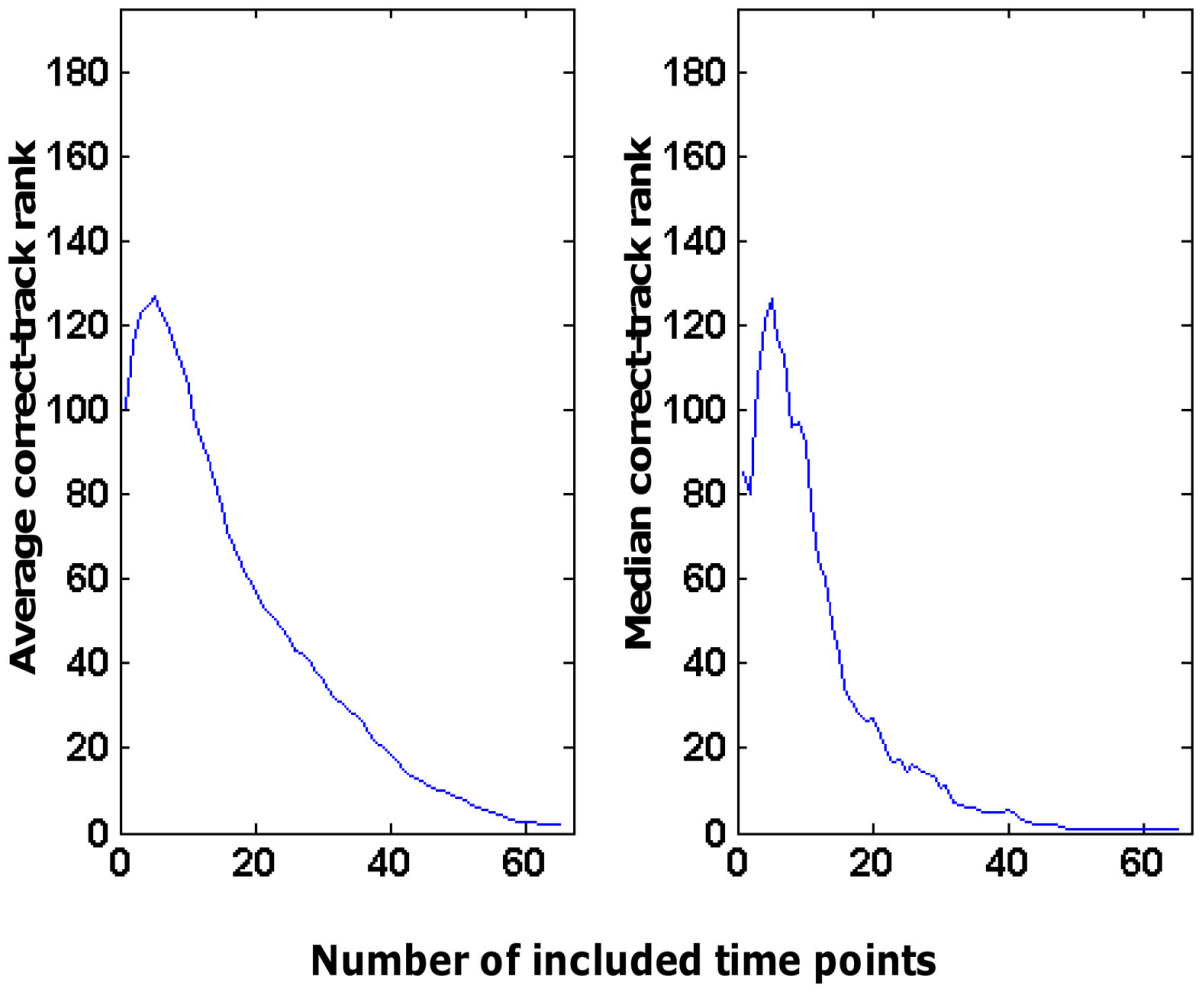
**Effect of the number of time points considered on the rank obtained by the correct track.** These data are based on the use of scene RMS information in combination with the V1 ROI to choose among 390 tracks (correct-track rank expected by chance is 196).

This performance was related to the fact that we combined information across time points: the more time points included, the better the correct-track rank. This is illustrated in Figure 6 for the decoding of tracks based on patterns of activity in area V1 related to scene RMS. This was the source × ROI combination that resulted in the best decoding performance overall. As was typical for all source × ROI combinations with significant decoding ability, the correct-track rank improved (lower rank) as a function of the number of time points. Sometimes there was a small bump with a low number of time points (also visible in Figure 6), which might be related to the fact that we always started with a very informative time point (start of a trial, often with full-screen viewing of a wall picture). However, overall the decoding of the route taken in a trial improved by adding time points.

Note that the different ROIs were not equal in size. Typically, pattern classification performance goes up with a larger number of voxels, which is the reason why we did not reduce all ROIs to the size of the smallest ROI (it would compromise decoding performance of the larger ROIs). It is important to note that our white-matter control region had a relatively large size, so the lack of decoding based on selectivity patterns in this region is not due to a small number of voxels. Thus, even with a large number of voxels, random data are shown to result in no decoding ability. As a result, the varying number of voxels across ROIs is not a problem for determining the statistical significance of our findings. Nevertheless, a small part of the variation in performance among the successful ROI × source combinations can be expected to be related to ROI size. As an extreme test, we reduced the largest ROI, V1 (1257 voxels), to 100 voxels, which is smaller than any of the ROIs tested (the 100 voxels were selected randomly). When combined with the scene RMS source of information, the normalized correct-track rank for this reduced V1 ROI was 0.21. This value is still much lower than the value expected by chance (0.50) or our criterion value for significance (0.32), but it has gone up compared to the normalized correct-track rank of 0.005 obtained with all V1 voxels.

### Combinatorial brain decoding: combining predictions across regions of interest and across sources of information

Decoding based on the data from individual ROIs and one specific source of information provided a ranking of the different alternative tracks according to how close the predicted values were to the actual values in each track. Here we combine these rankings of the different source × ROI combinations. Ranks were combined by a weighted average. Indeed, some source × ROI combinations were better than others, and these differences were very consistent across sessions/subjects (see Materials and Methods section). Thus, the source × ROI combinations that provided the best decoding were weighted most to come to a combined prediction. Decoding performance was expressed as the average correct-track rank (lower value is better). The weight of a source × ROI combination was an inverse linear function of this average correct-track rank so that rank 1 would give a weight of 1 and chance performance would result in a weight of 0.

When combining all sources of information, and all brain regions, the average correct-track rank was 1.07 when choosing among 390 alternative tracks. To characterize how good this performance is, we computed the percentage of the 106 fMRI trials that were picked out correctly (correct-track rank of 1) from the total database of 390 trials. Chance performance in this 1 out of 390 choice task is 0.3%. When combining all sources of information, and all brain regions, the percentage of correctly identified trials was 94% (correct performance on 100 out of 106 tracks).

Note that this successful decoding is based upon the combined effort of many classifiers. Given the number of SVM classifiers trained for each source of information for each ROI [also see Section General training and cross-validation procedure of support vector machine (SVM) classifiers], and given the number of source × ROI combinations with significant decoding, this accuracy of 94% is based upon the training of 203 classifiers.

The benefit of combining information across sources and ROIs is most obvious with sources × ROI combinations that were not close to perfect in isolation. If we leave V1 aside, which was the best ROI in many cases, the best performance on the 390-track problem was obtained by scene RMS combined with ventral visual cortex, giving an average correct-track rank of 13.50 (rank expected by chance is 196). If we combine all sources with all non-V1 ROIs, they nevertheless yielded an average correct-track rank of 1.48 (*SEM* across trials: 0.13). Thus, combining information across sources and ROIs helps significantly.

**Figure 7:**
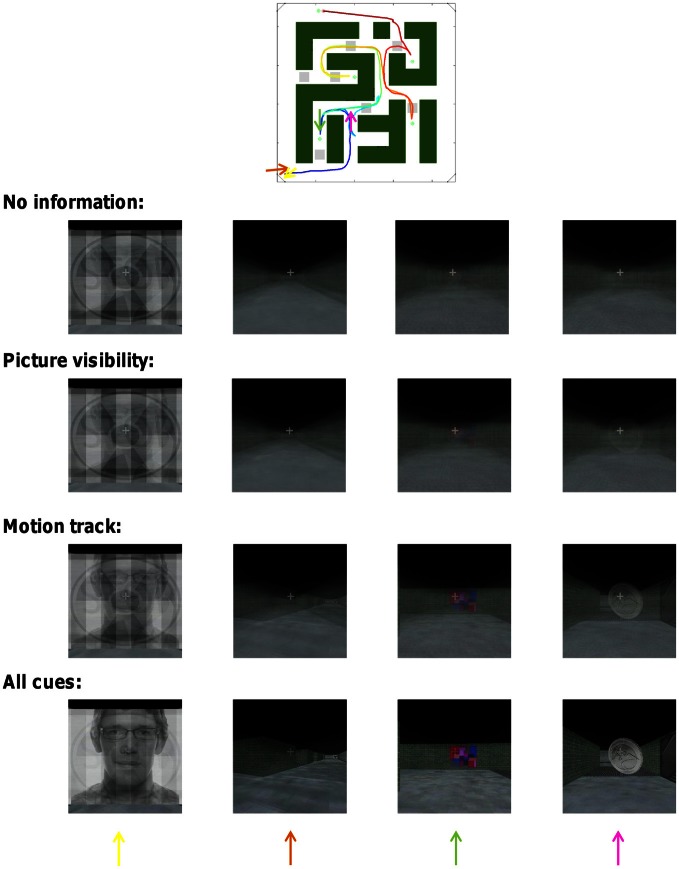
**Combinatorial decoding decreases the uncertainty about which track subjects have followed.** The impact of decoding upon uncertainty is illustrated visually for one track by the degree of averaging visual scenes across multiple tracks. Four time points of the track are shown (the arrows in the maze icon above correspond to the different columns). The exact face picture was changed for display purposes because the face picture in the actual experiment was purchased from a commercial website.

Average correct-track rank and the associated percentage of correct identification of the tracks is a quantitative but rather non-intuitive way of conveying the performance of our decoding approach. In Figure 7 we illustrate the performance of our decoding approach in a more visual way. Suppose we take the time period of one specific trial from our scanning data and consider the (un)certainty we have about which route subjects have followed during this time period. Without any information from brain activity, each of the alternative routes has an equal probability. In Figure 7 this is illustrated on the top row by averaging the scenes across the different routes with each route having an equal weighting. At the beginning of the track, when some of the pictures are seen at full-screen width, the scene is a mixture of those pictures. Later in the track, the guessed scene is very uninformative and constant: a blurry version of a straight and unidentifiable hallway.

Then we consider how much this uncertainty is reduced by what the decoding approach reveals about the probability of cue visibility. Using picture visibility information provided by all ROIs together, the performance of picking the correct track increases from the expected 0.3 to 8%. In Figure 7 this is illustrated on the second row by averaging the scenes across the different routes with the correct route having a weight of 8%. The uncertainty about the correct track is clearly reduced as some structure becomes apparent in the averaged image. The uncertainty is even more reduced based upon the motion track (third row of Figure 7), which is associated with a decoding performance of 30%. The uncertainty is finally reduced even more when based upon an integration of all sources of information and all ROIs which gave a decoding performance of 94% (Bottom row of Figure 7).

### Specific benefits of combining ROIs and/or sources of information

We performed more in-depth analyses to find out what type of combination helps most: integrating across ROIs that were all combined with the same source of information, or integrating across sources of information all combined with the same ROI. The result can be found in Table 1. The first column shows the source × ROI combinations that were integrated, followed by the average correct-track rank obtained by this integration, the standard error of this mean across the 106 trials, and finally the best correct-track rank that was achieved by using only one of the investigated single source × ROI combinations. Note that a rank of 196 would be chance performance as there were 390 tracks in our total pool of tracks.

Rows 2–6 in this table represent cases in which we integrated across ROIs that were all trained with the same source of information. Integrating across ROIs had a variable effect, and did not always result in better performance than the best single ROI. A very different picture emerged when we integrated across all sources of information combined with the same ROI (rows 7–9). The integrated correct-track rank, now with integration across sources, was always better than the best single-source case.

Why does it help to combine across sources of information? One hypothesis is that combining across sources of information makes decoding more robust against confusing tracks that are similar in one particular aspect. We tested this for tracks that were either similar in picture visibility or in motion path. Similarity to the correct track in motion path had a very strong effect on the rank of an incorrect track when the classifier providing that rank was trained using motion path information. When all ROIs are combined (cf. the weighted-average approach explained above), the rank of the five most motion-similar tracks was 57, compared to rank 333 for the five least similar tracks (difference significant across the 106 trials, paired *t*-test, *p* < 0.000001). No effect of similarity in motion path was present when decoding was based on picture visibility (correct-track rank of 181 vs. 193, *p* = 0.09).

A very different picture emerged when tracks were ranked according to how similar they are in terms of picture visibility. In this case, similarity in picture visibility had a strong effect on the rank of a track when classifiers were trained on picture visibility (tracks similar to correct track: 149; dissimilar tracks: 248, *p* < 0.000001), but smaller (albeit still significant with *p* = 0.0054) when classifiers were trained on motion path (similar tracks: 177; dissimilar tracks: 201). Thus, we have a double dissociation between the way in which tracks are similar and which sources of information will be most useful to differentiate the tracks. This illustrates the importance of using multiple sources of information for brain-based decoding of complex mental events.

## Discussion

We related the pattern of activity in multiple brain regions to the continuously changing position of subjects in a complex spatial environment. A direct decoding of a person's position in such an environment from the spatial map in hippocampus turned out to be difficult. Instead, we derived the route taken by subjects from patterns of fMRI activity in multiple brain regions that are related to sources of information which are only indirectly spatial. For example, pictures were hanging at fixed positions in the environment and thus the visibility of these pictures could potentially be used as a spatial cue. We found that picture visibility could be decoded based on patterns of activity in visual cortex, and the route taken by subjects could be derived, at least to some degree, from this decoded picture visibility. The same indirect decoding of spatial position also worked for other sources of information, including direction of movement and visual scene information. Decoding was far from perfect using single ROIs and single sources of information, but could be enhanced significantly by combining predictions across ROIs and sources of information. Many questions remain after this first study with a limited number of subjects, but it does serve as a “proof of principle” that it is in fact possible to decode detailed aspects of the behavior of subjects in very complex situations based on the dynamically changing pattern of activity in multiple brain regions, a methodology we refer to as “combinatorial brain decoding.”

The failure to decode spatial position directly from hippocampal activity is important in relative terms, in the sense that the direct decoding of spatial position is far worse relative to the indirect decoding of position from non-spatial information. This difficulty to decode spatial position directly is not in contrast with the literature. For example, several studies (Hassabis et al., 2009; Rodriguez, 2010) showed better-than-chance performance in a one-room spatial environment, but even in this very simple situation performance was already far from perfect. In a one-room environment, many hippocampal cells have a very simple place field that can be modeled by one preferred spot (strongest responses when the animal is at that spot) with a gradual decrease as distance increases from this preferred spot. With such a response profile it is relatively straightforward to derive spatial position from neural activity. However, in multiple-room environments, hippocampal cells typically do not code for the position of an animal in the overall environment. Instead, cells tend to have multiple place fields, with a preferred position in multiple rooms, and often the place fields of different rooms bear little relationship to each other (Derdikman and Moser, 2010). Such a spatial code is ill-suited to find out the overall position of a subject in a multiple-room environment using a simple decoding approach.

We must note, however, that our current study is very limited in terms of the number of subjects, that we only implemented relatively simple pattern classification techniques and one of many possible classification tasks (namely, a distinction in four quadrants), and that we did not perform a direct comparison of single-room vs. multiple-room environments. Further studies are needed before we can conclude with certainty that decoding of spatial position deteriorates as the complexity of the spatial environment increases. In addition our imaging parameters might not be optimal to pick up a reliable signal from hippocampus, e.g., scanning at higher resolution might have a beneficial effect. Nevertheless, it is clear that decoding spatial position in a complex spatial environment directly from fMRI patterns of activity that represent spatial information is less reliable than decoding spatial position indirectly from non-spatial sources of information.

There is a wealth of prior reports showing that fMRI patterns of activity are informative about many aspects of sensory and motor processing (Norman et al., 2006), including object category membership and shape properties in ventral visual cortex (e.g., Kriegeskorte et al., 2008; Op de Beeck et al., 2008; Drucker and Aguirre, 2009; Walther et al., 2009; Kravitz et al., 2011) and visual features in primary visual cortex (e.g., Kamitani and Tong, 2005; Kay et al., 2008; Swisher et al., 2010). The present study is the first in which the content of such non-spatial neural representations in multiple cortical regions is related to navigational behavior. Here we have mostly focused on decoding performance in order to highlight the potential of our decoding methods to provide a detailed picture of these representations. Nevertheless, this is only a first step, and future studies will have to include many additional manipulations in order to specify the exact nature of these representations.

Here one of the strengths of our approach, that we can investigate navigational events in their full complexity, is at the same time a weakness, in that we are limited in the way in which we can de-compose these navigational events into separate factors and cognitive processes. We identified multiple sources of information, but during navigation these sources of information are related to multiple factors that are confounded. For example, decoding of the motion track from V1 responses is expected to depend on visual processing, while decoding the same source of information from M1 responses is expected to reflect motor signals. While this result highlights the fact that during natural behavior most areas of the brain work together and convergence exists between the information encoded in very different brain systems, it does not take away from the fact that regions like V1 and M1 contain very different representations and are active for very different reasons. In this respect, the value of the current study lies mostly in the demonstration of the feasibility and high power of brain decoding fMRI to analyze behavior in very complex situations, providing the necessary basis for applying these methods in the context of further studies on the differences in what is represented in the multiple brain regions that are involved directly and indirectly in spatial navigation.

Because of the very different nature of the representations in regions such as V1 and M1, combining across ROIs representing the same source of information, which did not seem to help much in our current study, will have specific benefits that might be missed when focusing upon overall accuracy in the present study. In particular, combining across ROIs from different systems (e.g., visual and motor areas) will make decoding performance less susceptible to specific foils. For example, it might be difficult to differentiate passive viewing of a track from active navigation based on just visual regions, while the inclusion of motor cortex should make this task much easier. Thus, a multi-ROI approach would be necessary to avoid trivial pitfalls in the fMRI decoding of such complex events. Future studies that include such manipulations are needed to further pinpoint the specific content of neural representations during navigation.

In our approach, classifiers were developed for each individual ROI and classification results were combined a posteriori. This is a reasonable approach because ROIs differ in their decoding ability for different sources of information. For example, our approach allows us to only use motor cortex for those sources of information for which it contributes to decoding success. A different approach would be to use the voxels from all ROIs together and train classifiers with voxels from all ROIs, with in the limit a whole-brain approach. Such approach would necessitate the addition of feature extraction steps to the decoding, which would be an obvious extension of our methodology.

In sum, using combinatorial brain decoding we relate patterns of brain activity to the route followed by subjects in a complex maze environment. The decoding performance as shown here is most likely an under-estimation of the actual potential of our methods, because we have not yet explored further improvements, such as using different classifiers, specific feature extraction methods, improving spatial resolution, and more optimal approaches to weigh different cues and regions according to their reliability (e.g., Bayesian). Other interesting extensions could involve real-time decoding and combinations with other techniques such as EEG.

Even without these measures, our methods already significantly extend the potential of fMRI to decode complex natural thought during the execution of daily activities that typically engage multiple cognitive processes and brain regions. Rather than seeing these characteristics of natural behavior as a drawback for designing controlled experiments, we have adapted our methods in order to use these characteristics to our advantage by combining multiple sources of information, multiple brain regions, and multiple time points. Intriguingly, spatial representations in hippocampus and nearby cortex also integrate information across multiple sensory modalities and about self-motion, information that comes from multiple brain regions and that evolves dynamically over time (O'Keefe, 1976; Sharp et al., 1995; Andersen, 2007). From this perspective, the combinatorial methods that we developed in our study to decode how subjects navigate are a very crude version of how the brain itself constructs a cognitive spatial map of the environment and the subject's position.

### Conflict of interest statement

The authors declare that the research was conducted in the absence of any commercial or financial relationships that could be construed as a potential conflict of interest.

## Supplementary material

Movie S1 **The actual visual input received by the subject in the first part of the track shown in Figure 1B**. In order to reduce the size of this movie file, the sampling rate is 4 frames per s. The rate was 20 Hz during the actual experiment.
